# Longitudinal Trends of Participation in Relation to Mental Health in Children with and without Physical Difficulties

**DOI:** 10.3390/ijerph17228551

**Published:** 2020-11-18

**Authors:** Ai-Wen Hwang, Chia-Hsieh Chang, Mats Granlund, Christine Imms, Chia-Ling Chen, Lin-Ju Kang

**Affiliations:** 1 Graduate Institute of Early Intervention, College of Medicine, Chang Gung University, 259 Wen-Hwa 1st Road, Kwei-Shan, Tao-Yuan City 33302, Taiwan; awhwang@mail.cgu.edu.tw (A.-W.H.); clingchen@gmail.com (C.-L.C.); 2 Department of Physical Medicine and Rehabilitation, Chang Gung Memorial Hospital, Linkou, 5 Fu-Xing St., Kwei-Shan, Tao-Yuan City 33301, Taiwan; 3 Department of Pediatric Orthopedics, Chang Gung Memorial Hospital, Linkou, 5 Fu-Xing St., Kwei-Shan, Tao-Yuan City 33301, Taiwan; chiahchang@gmail.com; 4 CHILD, Swedish Institute of Disability Research, School of health and welfare, Jönköping University, Gjuterigatan 5, 553 18 Jönköping, Sweden; Mats.Granlund@ju.se; 5 Department of Paediatrics, The University of Melbourne, 50 Flemington Road, Parkville, Victoria 3052, Australia; christine.imms@unimelb.edu.au

**Keywords:** participation, longitudinal study, physical disabilities, inclusion, mental health

## Abstract

Children with physical disabilities (PD) are known to have participation restrictions when in inclusive settings alongside typically developing (TD) children. The restrictions in participation over time may affect their mental health status. This study aimed to investigate the longitudinal relationship between independence in activities (capability) and frequency of attendance in activities, in relation to perceived mental health status in children with and without PD. The participants were a convenience sample of parents of 77 school children with PD and 94 TD children who completed four assessments with a one-year interval between each assessment. Parents of these children were interviewed with the Functioning Scale of the Disability Evaluation System—Child version (FUNDES-Child). Three dimensions of mental health problems—loneliness, acting upset, and acting nervous—were rated by parents with the Child Health Questionnaire (CHQ). Linear trend was tested by repeated-measure ANOVA. The results revealed different longitudinal patterns of independence and frequency of attendance over time for children with PD and TD. Frequency of attending activities may be more important than independence in performing activities for experiencing fewer mental health problems. The findings highlight the need for supporting children’s actual attendance in daily activities which may benefit their later mental health.

## 1. Introduction

Participation, referring to functioning in everyday life beyond the health condition or disability-related diagnosis, is aligned with inclusive education in the Sustainable Development Goals (SDGs) as part of a United Nations Resolution that are intended to be achieved by 2030. SDGoal 4 states that inclusive and equitable quality education and promotion of lifelong learning opportunities in the home, school, and community “for all” must be ensured. Thus, children with disabilities have the same right to education and learning as other children. This SDG goal supports that all children should be educated within their best-fit environment, providing learning opportunities within participatory learning processes. Therefore, investigating whether the need for positive experiences and learning are met by the unique environmental requirements of children with disabilities will provide critical information for building a society for all.

Physical disability (PD) is one of the categories of disabilities defined in the overall objectives of the United Nations Convention on the Rights of Persons with Disabilities (UNCRPD). Typically, we address the need for safety and equality of school for children with physical difficulty with their peers in an inclusive physical environment [1]. However, the children’s mental health, especially in an inclusive setting, is usually not explicitly supported by the surrounding adults and peers. Mental health has been defined as “a state of wellbeing in which every individual realizes his or her own potential, can cope with the normal stresses of life, can work productively and fruitfully and is able to make a contribution to his or her community.” [2] School-aged children with physical disabilities [3] and young adults [4] are more likely to develop mental health problems, such as depression and anxiety, than their peers without disabilities.

Research reported increased vulnerability to poor mental health when adolescents make the transition to young adulthood [5]. The mental health of children with a physical disability aged 6–12 years is less well known. In Taiwan, caregivers and professionals focus largely on interventions to improve physical functioning, but mental health is seldom a focus of interest. However, the family costs associated with a mental disorder or mental illness are likely to be higher than those associated with chronic physical disorders [6]. 

Participation in everyday life activities can be seen as containing two dimensions; physical/virtual attendance and involvement [7]. The life situations in which these dimensions are experienced change over time which influences patterns of attendance and involvement. Long-term outcomes of attendance and involvement may with time affect mental health for children. Mental health may on the other hand affect the probability of adapting to environmental changes following from transition to new life roles. The two dimensions of participation have a bi-directional relation with internal factors within the child as well as external factors in the environment [7]. Internal factors concern activity competence, sense of self, and preferences, while external factors concern physical and social factors in the environment [7]. In earlier research and pediatric rehabilitation intervention, internal factors such as body functions and activity performance have been the focus with the implicit rational that by improving child skills the child will participate more. Thus, activity competence in terms of capability to perform activities in everyday life activities rather than participation in everyday life has been the focus of both assessment and intervention [8,9,10]. However, the evidence that intervention focusing solely on improving skills leads to enhanced participation is weak [9]. The relationship between activity competence, defined as capability, and the two dimensions of participation needs to be further investigated.

The physical and social activity competence of an individual can be investigated on a continuum from capacity (the ability to perform an activity under ideal circumstances) to capability (the ability to perform an activity in natural environments). In measures of activity competence, e.g., Pediatric Evaluation of Disability Inventory (PEDI) [11] or Child and Adolescent Scale of Participation (CASP) [12], activity competence is operationalized as independence, that is, the level of support needed to perform an activity. The Functioning Scale of the Disability Evaluation System—Child version (FUNDES-Child) [13,14] is a measure containing the further development of CASP to include a measure of frequency of attendance in activities, in addition to the measure of independence in performing an activity (capability). Thus, FUNDES-Child allows us to investigate the relationship between capability and the attendance dimension of participation.

We know that a low frequency of participation in physical activity can lead to a decrease in activity competence defined both as capacity and capability. The International Classification of Functioning, Disability and Health (ICF) framework has been applied in several longitudinal studies that indicate a bi-directional relationship between participation and body function (mental or physical) [15]. For children with severe physical impairments, the longitudinal prediction of participation by body function is stronger than for children with less severe physical impairments [16]. How mental health problems are related to capability as well as participation has been infrequently investigated. A cross-sectional study reported that participation in physical activities can attenuate the odds of depression in children with cerebral palsy (CP) (the Odds Ratio = 1.9; 95%; the Confidence Interval = 0.7–5.3) [3]. Another cross-sectional study reported a bidirectional relationship between mental health problems and participation for children with and without physical disabilities aged 6–14 years [17]. Studies are lacking about how capability and participation can predict or influence later mental health. 

It is likely that environmental factors moderate the relationship between capability and participation and mental health, respectively. Barriers in the environment may result in children with disabilities attending activities less frequently than same-aged peers, although they actually have the capability to perform the activity. Kang et al. found that barriers experienced in social support, such as attitudes from family and community, influenced participation more than the physical design of the school for children with physical disabilities [18]. Based on the reported difference between capability and frequency of attending an activity, Hwang et al. proposed that a measure of the gap between independence and frequency of attendance would reflect the closeness of fit between the environment and the person in relation to children with physical impairments [16]. A small gap would indicate a good fit. In Hwang et al.’s study, capability was defined as independence in performing an activity, and frequency of attendance was an operationalization of the attendance dimension of participation [19,20]. Hwang et al. explored the gap between independence and frequency using a nationwide cross-sectional survey with FUNDES-Child [16]. The data showed that the independence–frequency gap of children with cerebral palsy becomes wider with age and that the gap increased more for children with mild compared to severe impairments. The gap may reflect environmental and personal factors that influence individualized service plans or rehabilitation goals aimed at increasing the children’s attendance at activities even if they do not have the capability to perform the activities independently. 

Studies are needed to reveal the longitudinal influence of participation outcomes and its impacts on other outcomes. Imms and Adair (2016) in a longitudinal study investigated participation in activities outside the school for 93 children with CP for 9 years. Regarding attendance, the diversity of the activities the children attended, as well as the frequency with which the children attended the activities, decreased over time for recreational, active physical, and self-improvement activities; while attendance in social activities increased over time [21]. Anaby et al. (2019), in an intervention study aimed at increasing participation in community activities by adapting the environment, reported that increased self-rated perception of activity performance was related to increased motor capacity (a measure of activity competence) [22]. 

The purpose of this study was to investigate the longitudinal relationship between independence (capability) and frequency of attendance in relation to perceived mental health status in children with and without physical disabilities. Three specific aims were addressed to reveal the interactions between capability and attendance over time, and how these interactions relate to mental health status. First, the trajectories of independence, frequency of attendance, and the independence–frequency of attendance gap across four years were analyzed for children with and without physical disabilities. Second, the trajectories of independence, frequency of attendance, and the independence–frequency of attendance gap over time were compared in accordance with children’s mental health status. Third, the relationships between independence and frequency of attendance across the four years and mental health problems in the last year were examined.

## 2. Materials and Methods

### 2.1. Design

A four-year longitudinal descriptive study design was used. We analyzed data from children whose families completed surveys at four time points at one-year intervals. Trained interviewers visited each family to collect data.

### 2.2. Participants

The proxy–child dyads were recruited from elementary schools in the northern, middle, and southern parts of Taiwan. The inclusion criteria for children with physical disabilities were (1) children from the first to fifth grade; (2) children with the following primary diagnoses or conditions: Amputation, cerebral palsy, cerebral vascular accident/stroke (vascular brain disorders), congenital anomalies, hydrocephalus, juvenile arthritis, nonprogressive muscular disorders, neuropathy, orthopedic conditions (e.g., scoliosis), spinal cord injury, spina bifida, and traumatic brain injury [23], or those who had movement impairments [24] or neuromuscular disabilities [25]; and (3) that parents provided consent. The inclusion criteria for typically developing children were: (1) Children from the first to fifth grade; (2) children without medical diagnosis relating to developmental disabilities; and (3) that parents provided consent. The ethical approval (no. 100-4201A3) was obtained from the Institutional Review Board in the Chang Gung Memorial Hospital in Taiwan. All the participants provided the signed informed consent. The numbers of participants who completed the interviews from the first to the fourth time points were 119, 98, 97, and 94 for TD children, and 93, 78, 78, and 77 for children with PD, respectively (see Table 1 for demographic data). The attrition rates between the first and fourth time points were 21% for TD children and 17% for children with PD.

### 2.3. Measure

#### 2.3.1. Functioning Scale of the Disability Evaluation System—Child Version (FUNDES-Child)

The FUNDES-Child utilizes a proxy format in which parents or other caregivers answer questions about their child’s activities in the previous 6 months. The FUNDES-Child was translated and modified from the Child and Family Follow-up Survey (CFFS) [12]. The cross-cultural adaption and validation of FUNDES-Child has been reported elsewhere [13,14,18]. The FUNDES-Child contains four parts: Part I: Physical and emotional health (information on health and the way of moving and communication); Part II: Participation (derived from the Child and Adolescent Scale of Participation); Part III: Body function impairment (derived from the Child and Adolescent Factors Inventory); and Part IV: Environmental factors (derived from the Child and Adolescent Scale of Environment). In this study, we only focused on Parts I and II. General mental health status was measured by one question in the FUNDES-Child Part I, which was: “*In general, how would you describe your child’s emotional health and well-being (i.e., the way he or she feels about himself or herself and his or her life)?*” The response was rated as 0 (poor), 1 (fair), 2 (good), 3 (very good), and 4 (excellent). 

Participation was assessed using the FUNDES-Child Part II that contains 20 items of children’s daily participation in 4 settings: Home, neighborhood/community, school, and home/community living. The scale contains two dimensions: Independence and frequency of attendance [13]. Independence was defined as the chi1d’s current level of capability to perform the activity compared to other children of his or her age in the same community. For each item, independence was rated as 0 (independent), 1 (with supervision/mild assistance), 2 (with moderate assistance), 3 (with full assistance). Frequency of attendance was rated with reference to age as 0 (the same or more than expected for age), 1 (somewhat less than expected for age), 2 (much less than expected for age), and 3 (never does). The score was designed to match the coding of the ICF qualifiers, with higher scores indicating more limitations or restrictions in capability and performance. In the FUNDES-Child Part II (participation), therefore, a higher score for independence and frequency of attendance indicates a lower level of independence and a low frequency of attending in the activity. A response of “not applicable” (a child of the same age and in the same community would not be expected to do that activity) was allowed for both dimensions. For example, the item “using transportation to get around in the community” could be rated as not applicable if the child did not need to utilize the transportation system. All items were rated under the condition that children used assistive devices as usual. 

As each item in the FUNDES-Child Part II (participation) was on the same ordinal scale with the same anchor points at the extreme end (0–3 points), the two dimensions were comparable based on age-expected independence and frequency of attending. Items rated as “not applicable” were omitted in the scoring [13]. The mean scores for each of the 4 settings of FUNDES-Child Part II (participation) are thus the sum of the scores of all “applicable” items divided by the number of applicable items and then converted to a 0–100 scale for the two dimensions. The trained interviewers could, therefore, interpret the scores within the same directional framework (higher scores represented greater participation restriction and more dependence). A score of 0 on either scale could be interpreted as “doing the same as other children the same age”. The reliability of the FUNDES-Child Part II (participation) in children with and without physical disability was examined. Test–retest reliability of 86 parent proxies who were interviewed twice within 2 weeks was established for independence (intraclass correlation coefficient [ICC] = 0.955, *p* < 0.001) and frequency of attendance (ICC = 0.796, *p* < 0.001). Interrater reliability of another 77 parent proxy respondents was established for independence (ICC = 0.994, *p* < 0.001) and frequency of attendance (ICC = 0.860, *p* < 0.001).

#### 2.3.2. Child Health Questionnaire (CHQ)

The CHQ is an internationally recognized general health-related quality of life (HRQOL) instrument that has been rigorously translated into more than 78 languages and standardized for use with children aged 5–18 to assess the child’s physical, emotional, and social well-being. There are both parent-reported and child self-completed versions of varying lengths. This study applied the parent-reported 28 (PF28) version at the fourth time point of this study. The CHQ covers three items representing mental health problems: “*During the past 4 weeks, how much of the time do you think your child felt lonely?*”, “*During the past 4 weeks, how much of the time do you think your child acted nervous?*”, ”*During the past 4 weeks, how much of the time do you think your child acted bothered or upset?*” Each item is rated with the Likert scale as 1 (all of the time) to 5 (none of the time); thus, a higher score indicates less frequent mental health problems. The score was then transformed to standardized 0 to 100 scores using the algorithm *(raw score − 1)* × *100/4.* The higher standardized score means better mental condition. The whole scale score can be transformed to a Z-score as described in the manual [26]. 

### 2.4. Procedure

Study flyers and research invitations were distributed to schools and hospitals. The teachers and clinicians informed the researchers about the families who were interested in this study, and then the research assistants contacted the families. Following signed informed consent from the children’s proxies, the trained testers visited families at home or another place convenient to the family, such as schools or hospitals. The trained interviewers conducted structured interviews with the proxies to collect all data. To reduce participant attrition over time, thank you letters and an invitation for the next year were sent to participants’ schools and hospitals to be distributed to families every year around the time of Christmas or Chinese New Year.

### 2.5. Data Analysis

The independence–frequency of attendance gap was analyzed by the score of independence minus the score of frequency of attendance. If the independence–frequency of attendance gap was positive (i.e., independence limitation score > frequency of attendance restriction score, where high scores mean more dependent and restricted), it meant that children attended the activity more frequently than what was expected from their level of independence. If the gap was negative (i.e., independence limitation score < frequency of attendance restriction score), it meant that children attended the activity less frequently than what was expected from their level of independence. The trajectories of independence scores, frequency of attendance scores, and the independence–frequency of attendance gap were graphed to provide a global picture of the changes in patterns across four time points. The mean scores for independence and frequency were plotted on dual Y coordinates (from 0 “as expected for age” to 100 “most dependent” or “most restricted”, respectively), illustrating any discrepancy between independence and frequency of attendance scores.

Longitudinal statistical analyses were performed with the Statistical Package for Social Science version 21.0 (SPSS, Inc., Chicago, IL, USA). The changes from the first to the fourth year were examined with a two-way repeated measure ANOVA with a group (PD and TD) by time (the first, second, third, and fourth year) interaction. The trend analysis was performed with repeated measures of ANOVA. The significance of the linear trend was tested by ANOVA for 4 time points, and the between-times sum of squares for the effect of the time point was partitioned into a polynomial trend, namely a linear and higher order trend. The polynomial trend component was tested by an F-ratio (the mean square for linear trend/error term). To deal with the variance inequality, Leven’s test for homogeneity was conducted before ANOVA. Welch ANOVA and Games–Howell post hoc analyses were performed if the data failed to meet the equal variance assumption with alpha set at 0.05 (2-tailed). The trajectory of independence score, frequency of attendance score, and the independence–frequency of attendance gap were also graphed. 

To address the first and second aims, the above analyses were performed for all children with TD and PD and by the five levels of general mental health status (i.e., excellent, very good, good, fair, and poor) in each group. When analyzing each group based on the level of general mental health status, children with TD who were rated as “very good” and “good” and children with PD who were rated as “very good”, “good”, and “fair” had adequate sample sizes and thus sufficient statistical power for testing the significance in the trend analysis. For other children, only descriptive statistics were presented. To address the third aim, Pearson or Spearman correlations were used for examining the relationships between independence and frequency of attendance at the first to fourth time points and the mental health problems (i.e., loneliness, upset, and nervous) at the final time point. For exploratory purposes, we focused on correlations that reach a significance level of 0.05.

## 3. Results

**Figure 1:**
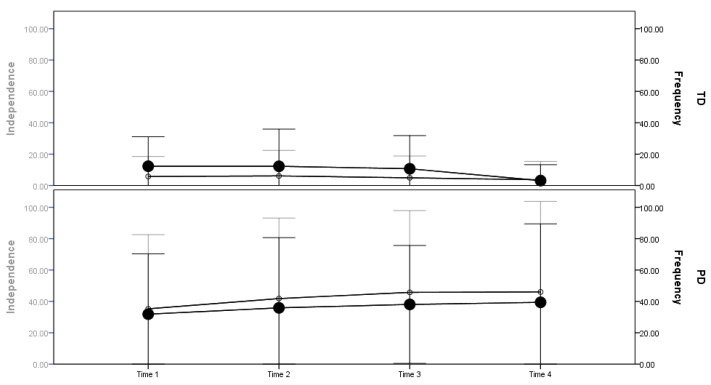
Independence and frequency of attendance gap by groups (TD vs. PD) across the four time points. Note: Dark point (●) and black line with 1 SD error bar illustrate the frequency of attendance scores; open circle point (○) and gray line with 1 SD error bar illustrate the independence scores.

The scores of independence and frequency of attendance measured by the FUNDES-Child Part II (participation) were significantly lower for children with PD than children with TD at each time point (*p* < 0.001). Patterns of change over the four time points showed that the children with PD had increasing scores (i.e., were more dependent and restricted) with age; while children with TD had decreasing scores (i.e., were less dependent and restricted) on the two dimensions (Table 2 and Figure 1). The independence–frequency of attendance gap scores for the TD children were initially negative; they tended to attend activities less frequently although they could perform the activity independently. With time the gap decreased and the change reached significance. For children with PD, the gap was positive, and they tended to be less dependent in the activity, although they attended the activity relatively frequently. With time, the gap increased but did not reach significance (Table 2).

**Figure 2:**
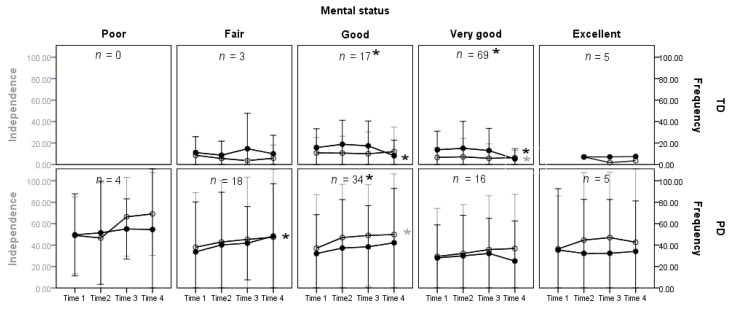
Independence and frequency of attendance gap by groups (TD vs. PD) across four time points by the five mental status of children. Note. TD = typically developing children; PD = physical disability; dark point (●) and black line with 1 SD error bar illustrate the frequency of attendance scores; circle point (○) and gray line with 1 SD error bar illustrate the independence scores; dark star sign (*) beside Time 4 presents a significant trend for frequency of attendance; gray star sign (*) beside Time 4 presents a significant trend for independence. Dark star sign (*) beside the case number presents significant independence–frequency of attendance gap trends in that block.

For the change patterns in independence and frequency of attendance across levels of general mental status, the children with PD had increasing scores (more limitations and restrictions) with time, while children with TD had decreasing scores (fewer limitations and restrictions; see Table 2 and Figure 2). The gap scores for the TD children were negative. With time, the gap decreased and the change reached significance for children whose mental health status was “very good” and “good”. For children with PD, the gap was positive. With time, the gap increased and the change reached significance only for children whose mental health status was “good” (Table 2). The interaction effects of time and group are available in the Supplementary Materials, Table S1.

**Figure 3:**
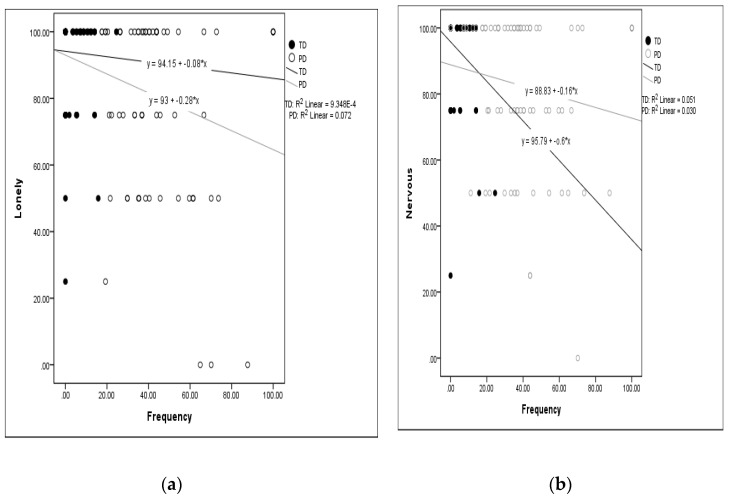
The scatter plots of correlations between (**a**) lonely and frequency; (**b**) nervous and frequency at the fourth time point.

The correlations between independence scores and frequency of attendance scores across the first to fourth time points and the items of mental health problems at the fourth time point are exhibited in Table 3. Overall, all correlations were in the weak-to-moderate range (<±0.4), and all but one was negative. Negative correlation coefficients between the independence and frequency of attendance scores and mental health problems scores indicate that more dependence and higher restrictions in attendance were associated with more mental health problems (i.e., lower scores for loneliness, upset, and nervous). In addition, the correlations between frequency of attendance and mental health problems were in general stronger than those for independence. The independence and frequency of attendance scores were correlated with the score for loneliness only for children with PD, and were also correlated with the score for being nervous only for children with TD. The frequency of attendance, but not independence, was correlated with the score for being upset for both children with TD and PD. When the scores of the three mental health items were aggregated to a Z-score, the frequency of attendance was correlated with the mental health scores at all 4 time points for children with PD, and also at second and third time points for TD children. The correlations between the frequency of attendance and loneliness and being nervous were highlighted by scatter plots of the scores at the fourth time (shown in Figure 3). The plots showed that the TD children who were more restricted in frequency of attendance expressed more feelings of being nervous. The children with PD who were more restricted in frequency of attendance expressed more loneliness.

## 4. Discussion

This study is unique for using a longitudinal design to investigate long-term changes in two dimensions, capability and frequency of attendance, and the closeness of fit between these two dimensions. This study described participation trajectories for the same group of children, providing strong evidence about their experiences and opportunities over time. The longitudinal investigation on participation for both children with PD and TD added valuable information to what can be deduced from cross-sectional data [16]. In a previous study, cross-sectional data of cohorts of children with PD showed a fluctuating and/or declining trend in participation attendance with age, especially during the transition from elementary school to junior high school [16]. With a longitudinal design, we were able to trace the adaptive process within the same groups of children across different ages. 

TD children on average had a decreasing negative gap between independence and frequency of attendance over time. In other words, with age, expected capacity (independence) and performance (participation) were matched. One explanation for this finding is that TD children experienced both increased capability and a stronger self-selection of what activities to attend frequently with age. In contrast, children with PD had decreasing independence over time and relatively stable frequency of attendance, suggesting that, despite a widening gap in age-appropriate independence, participation remained possible. Knowledge about typical and atypical trajectories could inform professionals in how to support children’s participation as a means of promoting both physical and mental health.

In terms of children’s general mental health status rated by parent proxies, a majority of children with TD were rated as good to very good; while a majority of children with PD were rated as fair to very good. This indicates that parents perceived a relatively good state of mental health status of school-age children in Taiwan. We would expect that when children with and without PD are in an inclusive environment, children with PD may need individualized strategies to enhance learning and socialization to a larger extent than TD children. Caregivers and educators may need supports in providing a learning and socially enhancing environment that helps children maintain an adequate level of mental health. A universal design for learning may be needed to meet the diverse participation needs that occur in inclusive education settings, thus supporting non-discriminatory and inclusive education. 

The trajectories identified for independence and frequency of attendance over four years were related to proxy-rated mental health status. Children with TD and PD who were less dependent and less restricted in attendance were also reported to have higher levels of mental health status. In particular, the highest dependence and restrictions in attending the activities were reported for children with PD rated as having poor mental health. However, the positive gap (i.e., attending more than the capability score suggested that a child could do independently) for children with PD remained over time, especially for children with good mental health. The positive gap may indicate the importance of support from the environment to enable frequent participation in the activities. For TD children, a gradual narrowing of the negative gap (due to sustained ability and increased frequency of attending an activity) was related to having good or very good mental health. At the last data collection point, TD children’s ability and frequency of attendance were matched. These findings suggest that school-age children with PD may have different lived experiences and adaptive processes from their TD peers as they age. For children with PD, continued environmental supports to enable children to attend more than their capability suggests may be an important support for maintaining good mental health status.

Though the children with disabilities have struggled with physical as well as emotional vulnerability, most children in this study were reported to have less frequent mental health problems, also indicating a relatively good state of mental health status. The relationship between frequency of attendance and later mental health problems was stronger than the relationship between independence and later mental health problems. Our results suggest that in children with PD, attending the activities more frequently was associated with less frequent feelings of loneliness, which is a positive outcome for social well-being. This suggests that it is essential to provide supports for children with PD to keep attending activities over time, regardless of whether they have limited ability to perform the activities independently. In children with TD, attending the activities more frequently was associated with less frequent feelings of nervousness. Experiences and competencies gained through participation may facilitate children’s confidence and mastery in performing the activities and is thus associated with positive emotional well-being [27].

Findings of this study have implications for environment-based interventions to achieve a match or even a positive gap between independence and frequency of attendance in activities for a child. Environment-based interventions focus on finding solutions built on the child’s strengths and capability that help to remove physical, social, and institutional or activity demands barriers to participation [15]. The active facilitation of participation for children with PD by adapting the environment may further facilitate the maintenance of children’s mental health. In particular, building a social-friendly environment relies on responsive relationships with care providers and teachers. For rights-based inclusion, the educator has a role to support the child to develop stable friendships by taking advantage of the positive characteristics of each child [28]. This would make inclusive education offer learning opportunities that engage every child, so they learn together and cope with each other.

This study highlighted the importance of exploring longitudinal patterns of capability and participation frequency in relation to general mental health status. There are, however, some limitations pertaining to the measures used in this study. In terms of mental health measures, only one item of general mental health status and three questions about mental health problems were used. Further research may explore a broader set of mental health issues that reflect emotional, psychological, and social well-being. In terms of the measures of participation, children’s involvement in the activities was not investigated. It is likely that personal feelings and experiences when actually engaging in the activities are affecting children’s mental health and well-being. The relationship between involvement and mental health warrants further investigation.

## 5. Conclusions

Children with physical disabilities can, presumably with appropriate supports, sustain a high frequency of attending activities despite difficulties with performing the activities independently. Enriched participation experiences may lead to better mental status of children regardless of disability or not. However, the relationships between frequency of attending, independence, and mental health differed between children with and without PD. Children with TD exhibited fewer mental health problems as rated by proxies, and their negative frequency of attending–independence gap narrowed over time. Children with PD still had a wide positive gap after four years of life experiences. Loneliness was related to less frequent attendance for children with PD, while acting nervously was related to less frequent attendance for children with TD. Interventions for promoting mental health status may be designed based on universal strategies that support participation as well as the characteristics of the individual child.

## Figures and Tables

**Table 1:** The demographic data for children with typical development (TD) and children with physical disabilities (PD).

	TD (*n* = 94)				PD (*n* = 77)			
	Time 1	Time 2	Time 3	Time 4	Time 1	Time 2	Time 3	Time 4
Gestational age (weeks) (SD)	37.5 (2.8)				33.5 (5.4) ^a^			
Age (months) (SD)	95.6 (17.7)	109.1 (17.7)	122.9 (18.3)	135.6 (17.9)	92.5 (17.1)	109.2 (19.2)	122.2 (19.7)	134.9 (19.9)
Gender (male) (N, %)	46 (50.5)				53 (68.8) ^b^			
Major Diagnosis ^c^								
CP					46 (59.7)			
Seizure					16 (20.8)			
Hydrocephalus					3 (3.9)			
Brain hemorrhage					1 (1.3)			
CHD					5 (6.5)			
Down syndrome					1 (1.3)			
Rare disease					1 (2.3)			
GMFCS ^d^								
I					7 (15.2)			
II					9 (19.6)			
III					13 (28.2)			
IV					10 (21.7)			
V					7 (15.2)			
MACS ^d^								
I					6 (13)			
II					15 (32.6)			
III					8 (17.4)			
IV					13 (28.3)			
V					4 (8.7)			
CFCS ^d^								
I					20 (43.5)			
II					8 (17.4)			
III					9 (19.6)			
IV					7 (15.2)			
V					2 (4.3)			
School grade (N, %)								
Kindergarten	14 (15.4)	1 (1.1)	0 (0)	0 (0)	14 (18.2)	1 (1.3)	3 (3.9)	2 (2.6)
First grade	20 (22)	10 (11)	1 (1.1)	0 (0)	23 (29.9)	14 (18.2)	0 (0)	1 (1.3)
Second grade	28 (30.8)	19 (20.9)	9 (9.9)	2 (2.2)	17 (22.1)	17 (22.1)	13 (16.9)	0 (0)
Third grade	19 (20.9)	25 (27.5)	18 (19.8)	8 (8.8)	16 (20.8)	14 (18.2)	15 (19.5)	13 (16.9)
Fourth grade	6 (6.6)	17 (18.7)	26 (28.6)	19 (20.9)	6 (7.8)	17 (22.1)	16 (20.8)	15 (19.5)
Fifth grade	2 (2.2)	15 (16.5)	16 (17.6)	25 (27.5)	1 (1.3)	11 (14.3)	17 (22.1)	15 (19.5)
Sixth grade	2 (2.2)	2 (2.2)	17 (18.7)	16 (17.6)	0 (0)	2 (2.6)	10 (13)	18 (23.4)
Seventh grade		2 (2.2)	2 (2.2)	17 (18.7)		1 (1.3)	3 (3.9)	10 (13)
Eighth grade		2 (2.2)	2 (2.2)	2 (2.2)		0 (0)	3 (3.9)	3 (3.9)
Ninth grade				2 (2.2)				0 (0)
School placement (N, %)								
Regular	94 (100)				15 (20.3)	13 (18.1)	17 (22.1)	9 (13.8)
Regular and resource room	0 (0)				20 (27)	23 (31.9)	22 (28.6)	18 (27.7)
Special class in regular school	0 (0)				25 (33.8)	30 (41.7)	30 (39)	26 (40)
Special school	0 (0)				4 (5.4)	4 (5.6)	6 (7.8)	8 (12.3)
Residential school or home	0 (0)				1 (1.4)	1 (1.4)	1 (1.3)	2 (3.0)
Others	0 (0)				9 (12.2)	1 (1.4)	1 (1.3)	2 (3.1)

SD = standard deviation; CP=cerebral palsy; CHD=Congenital Heart Disease. ^a^ Significant difference between children with TD and PD by independent *t*-test, alpha set at 0.05; ^b^ significant difference between children with TD and PD by chi-square, alpha set at 0.05; ^c^ children’s major diagnoses were obtained from physicians; ^d^ only children with CP were rated by interviewers with the Gross Motor Function Classification System (GMFCS), Manual Ability Classification System (MACS), and the Communication Function Classification System (CFCS) at the first time point test.

**Table 2:** Results of longitudinal statistical analyses for children with PD and TD children.

	TD						PD					
	Mean (SD)				Trend		Mean (SD)				Trend	
	Time 1	Time 2	Time 3	Time 4	(df = 3)	*p*	Time 1	Time 2	Time 3	Time 4	F	*p*
FUNDES-Child total scores for whole participants with typical development (*n* = 94) and children with disabilities (*n* = 77).												
Independence	5.74 (6.37)	6.12 (8.14)	4.89 (6.93)	3.65(5.87)	5.575	0.019	35.28 (23.62)	41.80 (25.68)	45.75 (26.09)	46.01 (28.87)	7.367	0.007
Frequency	12.27 (9.42)	12.25 (11.86)	10.68 (10.57)	3.12 (5.08)	43.193	<0.001	31.85 (19.23)	35.89 (22.37)	38.05 (18.80)	39.39 (25.03)	5.112	0.024
Gap	−6.54 (8.77)	−6.14 (7.97)	−5.79 (7.78)	0.53 (4.00)	40.202	<0.001	3.43 (16.25)	5.92 (15.99)	7.70 (16.66)	6.62 (14.45)	1.978	0.161
Mental health excellent for children with typical development (*n* = 5) and children with disabilities (*n* = 5) (NA).												
Mental health very good for children with typical development (*n* = 69) and children with disabilities (*n* = 16).												
Independence	5.11 (6.04)	5.41 (7.66)	4.62 (6.51)	2.98 (3.95)	4.648	0.032	27.81 (22.56)	30.31 (23.29)	33.83 (25.53)	34.53 (26.06)	0.753	0.389
Frequency	12.73 (9.71)	11.79 (11.45)	10.40 (10.28)	2.64 (4.28)	39.655	<0.001	26.71 (15.76)	28.09 (19.76)	30.78 (16.83)	23.52 (19.14)	0.117	0.733
Gap	−7.62 (9.55)	−6.39 (8.72)	−5.78 (7.26)	0.34 (3.39)	35.810	<0.001	1.10 (17.57)	−6.39 (8.72)	−5.78 (7.26)	0.34 (3.39)	2.910	0.093
Mental health good for children with typical development (*n* = 17) and children with disabilities (*n* = 34).												
Independence	8.34 (7.41)	9.44 (10.32)	7.26 (9.36)	7.04 (10.49)	0.351	0.556	37.06 (24.99)	47.06 (24.81)	49.00 (23.67)	49.79 (28.27)	4.213	0.042
Frequency	12.91 (8.44)	16.60 (14.49)	13.64 (11.36)	4.79 (6.85)	5.559	0.021	32.10 (18.13)	37.25 (22.61)	38.40 (19.16)	42.12 (25.37)	3.577	0.061
Gap	−4.57 (5.37)	−7.16 (5.4)	−6.38 (8.73)	2.25 (4.76)	1.380	0.003	4.96 (18.22)	9.81 (15.02)	10.61 (17.62)	7.67 (14.96)	0.496	0.482
Mental health fair for children with typical development (*n* = 3) and children with disabilities (*n* = 18).												
Independence	8.51 (8.67)	5.56 (8.07)	3.51 (0.01)	5.85 (6.16)	NA	NA	35.20 (23.00)	40.24 (27.11)	45.30 (29.06)	44.91 (30.69)	0.138	0.244
Frequency	11.11 (7.41)	8.64 (6.50)	14.65 (16.54)	9.94 (8.65)	NA	NA	31.05 (20.86)	37.81 (22.85)	41.69 (17.09)	46.45 (23.56)	5.008	0.029
Gap	−2.60 (4.33)	−3.09 (5.35)	−11.14 (16.54)	−4.09 (10.72)	NA	NA	4.15 (14.56)	2.44 (13.2)	3.61 (17.23)	−1.55 (15.21)	0.997	0.332

Note: Mental health poor for children with typical development (*n* = 0) and children with disabilities (*n* = 4) (NA).

**Table 3:** Correlation coefficients between mental health problems measured by the Child Health Questionnaire (CHQ) and participation independence and frequency of attendance.

	T4 Lonely		T4 Upset		T4 Nervous		Z-Score	
	TD	PD	TD	PD	TD	PD	TD	PD
T1 Independence	0.025	−0.169	−0.051	−0.142	−0.114	−0.165	−0.050	−0.179
T2 Independence	−0.104	−0.169	−0.172	−0.124	−0.248 *	−0.064	−0.186	−0.137
T3 Independence	−0.072	−0.288 *	−0.139	−0.201	−0.284 **	−0.124	−0.176	−0.235 *
T4 Independence	−0.003	−0.258 *	−0.175	−0.183	−0.301 **	−0.099	−0.170	−0.207
T1 Frequency	−0.029	−0.167	−0.031	−0.231 *	−0.046	−0.204	−0.038	−0.225 *
T2 Frequency	−0.121	−0.332 **	−0.209 *	−0.178	−0.267 **	−0.095	−0.212 *	−0.233 *
T3 Frequency	−0.184	−0.342 **	−0.225 *	−0.281 *	−0.358 **	−0.147	−0.273 **	−0.294 **
T4 Frequency	−0.039	−0.268 *	−0.133	−0.207	−0.232 *	−0.174	−0.144	−0.246 *

* *p* < 0.05; ** *p* < 0.01; T1 to T4 stand for the first to fourth time points.

## Supplementary Materials

The following are available online at https://www.mdpi.com/1660-4601/17/22/8551/s1, Table S1: Main and interaction effects of longitudinal statistical analyses for children with PD and TD children.

## Abbreviations

TD	Typical development
CP	Cerebral palsy
CHD	Congenital heart disease
GMFCS	Gross Motor Function Classification System
MACS	Manual Ability Classification System
CFCS	Communication Function Classification System
