# Supplementary material for: Longitudinal Trends of Participation in Relation to Mental Health in Children with and without Physical Difficulties
Source: Int J Environ Res Public Health. 2020 Nov 18;17(22):8551. doi: 10.3390/ijerph17228551 (PMC7698882; doi:10.3390/ijerph17228551)
Supplement: Supplementary file 1 [file ijerph-17-08551-s001.pdf]

Table S1. Main and interaction effects of longitudinal statistical analyses for children with PD and TD children.

	TD			PD			Main effect [b]				Interaction	
		Trend [a]			Trend [a]		Time		Group			
	Time 1 to Time 4	(df = 3)	*p*	Time 1 to Time 4	F	*p*	F, (df = 3)	P	F, (df = 1)	P	F, (df = 3)	P
FUNDES-child total scores for whole participants with typical development (*n* = 94) and children with disabilities (*n* = 77)												
Independence	Capability increased (scores down trend)	5.575	0.019	Capability decreased (scores up trend)	7.367	0.007	10.470	<0.001	205.87	<0.001	17.359	<0.001
Frequency	Attendance increased (scores down trend)	43.193	<0.001	Attendance decreased (scores up trend)	5.112	0.024	3.508	0.017	178.56	<0.001	15.521	<0.001
Gap	Narrowing trend	40.202	<0.001	Non-significant widening trend	1.978	0.161	7.600	<0.001	62.781	<0.001	5.291	0.002
Mental Health excellent for children with typical development (*n* = 5) and children with disabilities (*n* = 5) (NA)												
Mental Health very good for children with typical development (*n* = 69) and children with disabilities (*n* = 16)												
Independence	Capability increased (scores down trend)	4.648	0.032	Capability decreased (scores non-sig up trend)	0.753	0.389	1.680	0.178	88.810	<0.001	5.622	0.001
Frequency	Attendance increased (scores down trend)	39.655	<0.001	Non-sig and non-linear trend	0.117	0.733	10.091	<0.001	54.276	<0.001	1.735	0.166
Gap	Narrowing trend	35.810	<0.001	Non-sig narrowing trend	2.910	0.093	13.571	0.000	23.369	<0.001	0.215	0.886
Mental Health good for children with typical development (*n* = 17) and children with disabilities (*n* = 34)												
Independence	Capability increased (scores non-sig down trend)	0.351	0.556	Capability decreased (scores up trend)	4.213	0.042	3.630	0.019	39.953	<0.001	4.493	0.007
Frequency	Attendance increased (scores down trend)	5.559	0.021	Attendance decreased (scores non-sig up trend)	3.577	0.061	1.327	0.277	35.934	<0.001	3.475	0.023
Gap	Narrowing trend	1.380	0.003	Non-significant widening trend	0.496	0.482	1.353	0.269	17.054	<0.001	3.196	0.032
Mental Health fair for children with typical development (*n* = 3) and children with disabilities (*n* = 18)												
Independence	NA	NA	NA	Capability decreased (scores non-sig up trend)	0.138	0.244	NA	NA	NA	NA	NA	NA
Frequency	NA	NA	NA	Attendance decreased (scores up trend)	5.008	0.029	NA	NA	NA	NA	NA	NA
Gap	NA	NA	NA	Non-sig narrowing trend	0.997	0.332	NA	NA	NA	NA	NA	NA
Mental Health poor for children with typical development (*n* = 0) and children with disabilities (*n* = 4) (NA)												

Note. PD: physical disability; TD: typically developed; GAP: independence-frequency of attendance gap; NA: statistics not appropriate. [a] The trend analysis was performed with repeated measures ANOVA, followed by Scheffé's post hoc analyses. To deal with the variance inequality, Leven’s test for homogeneity was conducted before ANOVA. Welch ANOVA and Games-Howell post hoc analyses were performed if the data failed to meet the equal variance assumption with alpha set at 0.05 (2-tailed). [b] The changes from the 1st to the 4th year were examined with Two-way ANOVA with repeated measure with group (PD and TD) by time (the 1st, 2nd, 3rd and 4th year) interaction.
